# Mitochondrial dysfunction within the synapses of substantia nigra neurons in Parkinson’s disease

**DOI:** 10.1038/s41531-018-0044-6

**Published:** 2018-03-26

**Authors:** Amy K. Reeve, John P. Grady, Eve M. Cosgrave, Emma Bennison, Chun Chen, Philippa D. Hepplewhite, Christopher M. Morris

**Affiliations:** 1 MRC/BBSRC Centre for Ageing and Vitality and Wellcome Centre for Mitochondrial Research, Institute for Neuroscience, Newcastle University Institute for Ageing, Newcastle University, Newcastle upon Tyne, NE2 4HH UK; 2 Kinghorn Centre for Clinical Genomics, Garvan Institute, 384 Victoria Street, Darlinghurst, Sydney NSW 2010 Australia; 3 Newcastle Brain Tissue Resource, Newcastle University, Edwardson Building, Campus for Ageing and Vitality, Newcastle upon Tyne, NE4 5PJ UK; 4 Department of Cellular Pathology, Royal Victoria Infirmary, Queen Victoria Road, Newcastle upon Tyne, NE1 4LP UK; 5 Medical Toxicology Centre, Wolfson Building, Claremont Place, Newcastle upon Tyne, NE2 4AA UK

## Abstract

Mitochondrial dysfunction within the cell bodies of substantia nigra neurons is prominent in both ageing and Parkinson’s disease. The loss of dopaminergic substantia nigra neurons in Parkinson’s disease is associated with loss of synapses within the striatum, and this may precede neuronal loss. We investigated whether mitochondrial changes previously reported within substantia nigra neurons were also seen within the synapses and axons of these neurons. Using high resolution quantitative fluorescence immunohistochemistry we determined mitochondrial density within remaining dopaminergic axons and synapses, and quantified deficiencies of mitochondrial Complex I and Complex IV in these compartments. In Parkinson’s disease mitochondrial populations were increased within axons and the mitochondria expressed higher levels of key electron transport chain proteins compared to controls. Furthermore we observed synapses which were devoid of mitochondrial proteins in all groups, with a significant reduction in the number of these ‘empty’ synapses in Parkinson’s disease. This suggests that neurons may attempt to maintain mitochondrial populations within remaining axons and synapses in Parkinson’s disease to facilitate continued neural transmission in the presence of neurodegeneration, potentially increasing oxidative damage. This compensatory event may represent a novel target for future restorative therapies in Parkinson’s disease.

## Neurodegeneration: powering communication in the face of adversity

Large numbers of healthy mitochondria are found along neuronal axons in postmortem brain tissue from patients with Parkinson’s disease (PD). Mitochondrial dysfunction is implicated in neurodegenerative diseases and is thought to precede neuronal loss. Although disrupted mitochondria have been reported in neuronal cell bodies, little is known about the mitochondria in axons and at synapses of substantia nigra neurons in PD. Amy Reeve at Newcastle University, UK, and colleagues found that although PD neurons have fewer synapses, their axons contained more mitochondria than age-matched controls. Moreover, there were fewer synapses devoid of mitochondria than in controls. Given that mitochondria power the movement and recycling of neurotransmitter containing vesicles at synapses, these findings suggest that in PD neurons attempt to compensate for the loss of synapses by maintaining neurotransmission in the remaining ones.

## Introduction

Parkinson’s disease (PD) is classically associated with a loss of dopaminergic substantia nigra (SN) neurons, however it may be the progressive loss of their synapses and axons that leads to the depletion in dopamine neurotransmission.^1^ Profound SN neuron loss is seen in longstanding PD, although around 70% of SN neurons need to be lost before clinical symptoms are evident,^2^ therefore either there is considerable redundancy within the SN projection to the striatum or there is compensatory synaptic sprouting as degeneration takes hold. Striatal dopamine may be depleted by up to 80% by the time of onset of motor symptoms and imaging has shown that changes in dopamine transporter (DAT) expression may be helpful for monitoring of progression^3^ and the detection of changes in at-risk individuals.^4,5^

Many genes associated with familial PD also cause synaptic dysfunction when mutated or down regulated. Perhaps the most prominent of these is the alpha-synuclein gene, SNCA.^6^ A relatively small and unstructured protein, alpha-synuclein has been proposed to have several functions at the synapse, including modulation of dopamine release and a regulation of ion channels.^7^ Alpha-synuclein is also capable of interacting with mitochondria and causing their dysfunction,^8^ indeed it has been shown that alpha-synuclein can induce mitochondrial dysfunction within SN neurons that precedes neuronal loss.^9–11^ Furthermore, knock-down of Pink1, Parkin or DJ-1 causes synaptic dysfunction, with reduced dopamine release and synaptic plasticity in the striatum.^12–15^ Similarly, LRRK2 has been shown to interact with a number of synaptic proteins to affect the mobilisation of synaptic vesicles,^16^ and mutations in LRRK2 cause a reduction in dopamine release into the striatum.^17–19^

Mitochondrial dysfunction has also been linked to SN neuronal loss and can take the form of reduced electron transport chain protein expression and activity,^20–22^ which may be caused by the accumulation of mitochondrial DNA deletions.^22,23^ Pink1, Parkin and DJ-1 encode proteins with essential mitochondrial functions and their disruption can cause marked changes in the function and ultrastructure of mitochondria.^13,24–29^ Furthermore, MPTP treatment causes a loss of dopaminergic synapses followed by SN cell bodies; however protection of the synaptic terminals against oxidative stress prevents the subsequent loss of SN neurons.^30,31^ Electron microscopy of dopaminergic synapses within the caudate nucleus of PD patients has revealed an increase in the synaptic area occupied by mitochondria, however mitochondrial function was not examined.^32^ More recent data has suggested that dysfunctional mitochondria may be transported retrogradely to the cell body for lysosomal degradation, thus ensuring that the synapse and axon are populated by healthy mitochondria with intact respiratory activity (reviewed in ref. ^33^). However it remains unclear whether mitochondrial deficiencies are also seen within dopaminergic axons and synapses and how they affect the metabolically demanding process of synaptic transmission.^34^ Here we investigated the contribution of synaptic changes and mitochondrial dysfunction to the neurobiology of PD. We measured the level of mitochondrial dysfunction and volume within single synapses and axons of SN neurons to ascertain whether mitochondrial dysfunction is differentially distributed within these neuronal compartments and the impact of this on synaptic morphology and potential function.

## Results

### Changes in synaptic volume in Parkinson’s disease

**Fig. 1:**
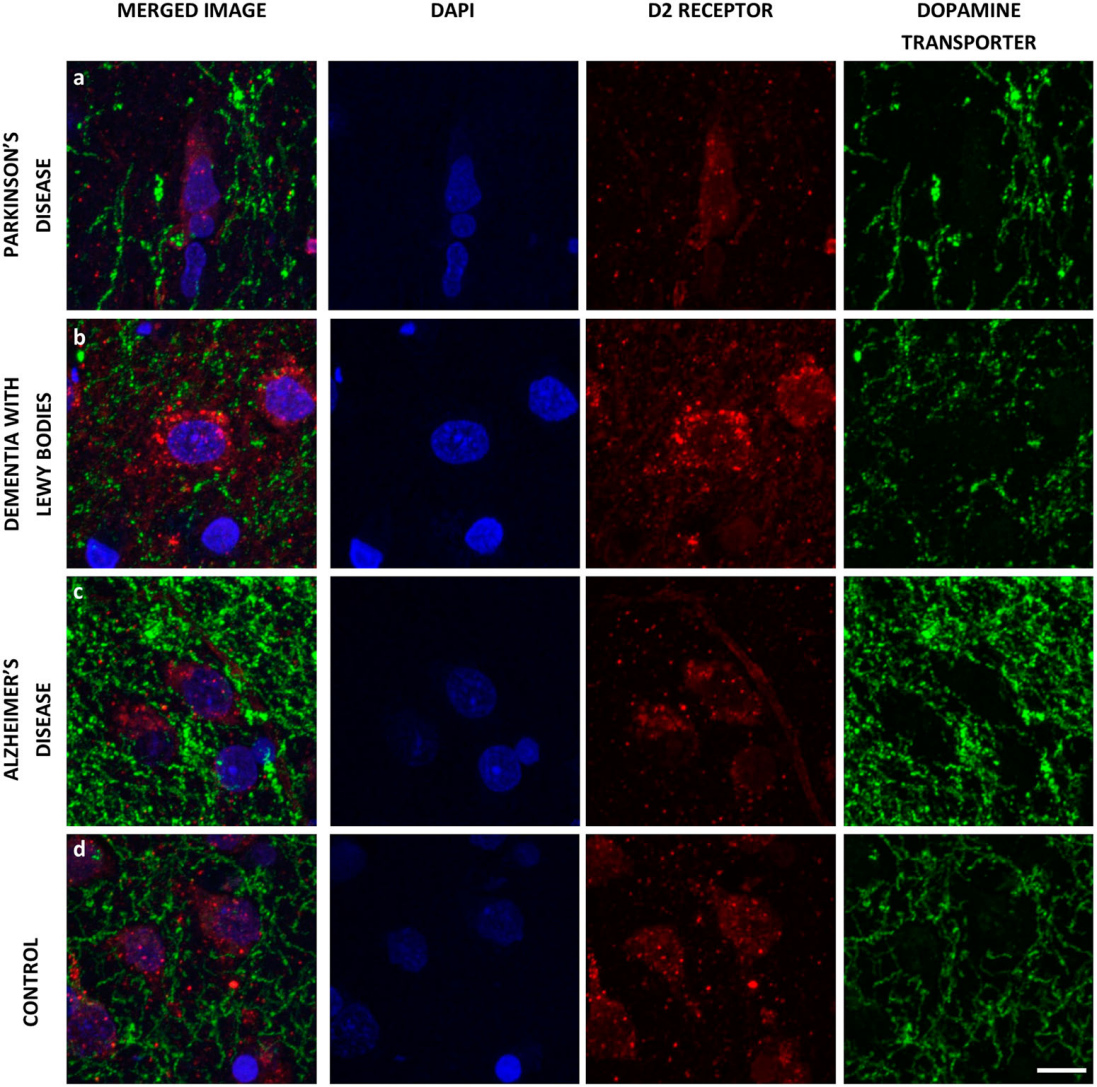
Representative images of pre and post synaptic immunoreactivity. Synaptic terminals of dopaminergic SN neurons within the striatum were analysed. Pre-synaptic terminals were visualised using reactivity for the dopamine transporter, DAT (green, 488 nm). Images were taken of pre-synaptic terminals surrounding a post synaptic neuron, post synaptic regions were visualised using reactivity for the dopamine D2 receptor (red, 546 nm). Pre and post synaptic volume was measured in tissue from patients with PD (**a**), DLB (**b**), AD (**c**) and in age matched controls (**d**). Scale bar represents 10 µm

**Fig. 2:**
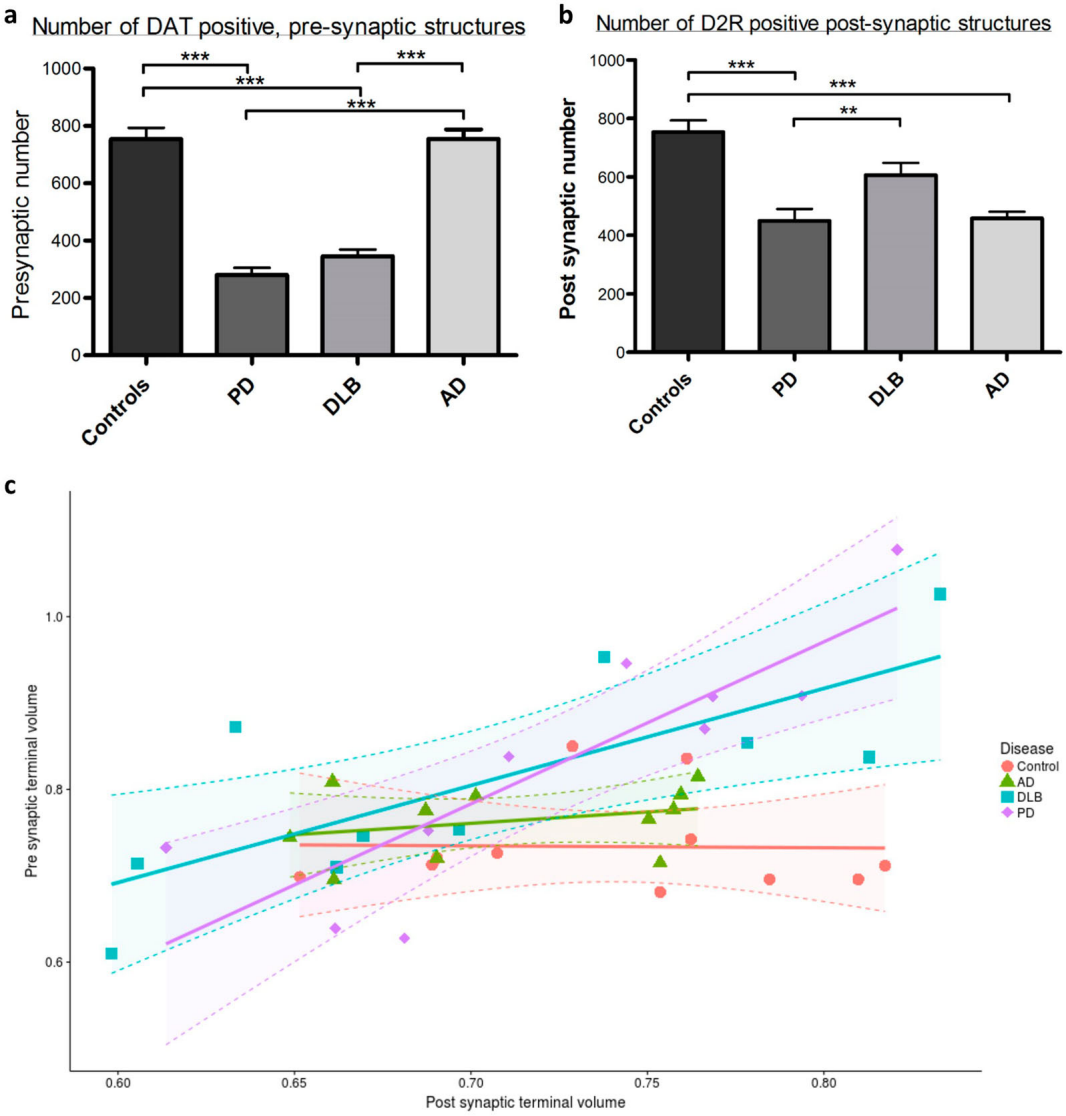
Changes in synaptic volume and density are detected in PD cases. Synaptic loss has been shown to occur in Parkinson’s disease, and has been thought to precede neuronal loss within the substantia nigra. The immunofluorescent assay used in this study and the subsequent image analysis could detect this loss of synapses within the striatum of patients affected by PD and DLB. There was a significant reduction in pre-synaptic DAT positive terminals in PD and DLB compared to controls and AD (***p = ≤0.001) (**a**). Interestingly we saw an associated loss of post-synaptic D2R positive structures in PD and AD, but not in DLB (***p = ≤0.001; **p = ≤ 0.01) (**b**). Kruskal–Wallis one-way ANOVAs were performed with Dunn’s multiple comparison testing to ascertain statistical significance. A significant increase in synaptic volume was found to occur in PD and (**c**) shows the relationship between pre and post synaptic structure volume in all four patient groups. The volume of pre and post synapses was only found to be related in PD (*p* = 5 × 10^−6^) and DLB (*p* = 0.0002). Error bars represent s.e.m

To ascertain whether any structural changes occurred within synapses of substantia nigra neurons we measured the volume of both pre-synaptic terminals and their corresponding post-synaptic region. We employed dual immunofluorescence for the dopamine transporter (pre synaptic terminal) and the dopamine D2 receptor (post synaptic region) (Fig. 1) and the number of immunoreactive objects per image was then calculated. We detected, as expected, a significant reduction in pre-synaptic DAT positive terminals within the putamen in PD and dementia with Lewy bodies (DLB) cases, compared to controls and AD cases (*p* ≤ 0.001, *n* = 6 images analysed per case, Fig. 2a). Interestingly we also detected a loss of D2R positive, post synaptic terminals in PD and AD cases (*p* ≤ 0.001, *n* = 6 images analysed per case) compared to controls and in PD cases compared to DLB cases (*p* ≤ 0.01, *n* = 6 images analysed per case) (Fig. 2b).

The volume of dopaminergic terminals within the striatum was measured. This showed that there was a significant relationship between the size of the pre and post-synaptic structures detected in PD and DLB cases (*p*-values are <0.0001 and 0.0002, respectively), based on the gradients of regression lines (Fig. 2c), but not in the AD (*p* = 0.577) or control groups (*p* = 0.957). Furthermore there was a trend for the pre-synaptic volume to be increased in PD and DLB compared to controls, which reached significance in the group analysed for synaptic volume alongside NDUFB8 and porin (PD *p* = 0.0034; DLB *p* = 0.005).

### Mitochondrial alterations in surviving dopaminergic axons within the striatum

**Fig. 3:**
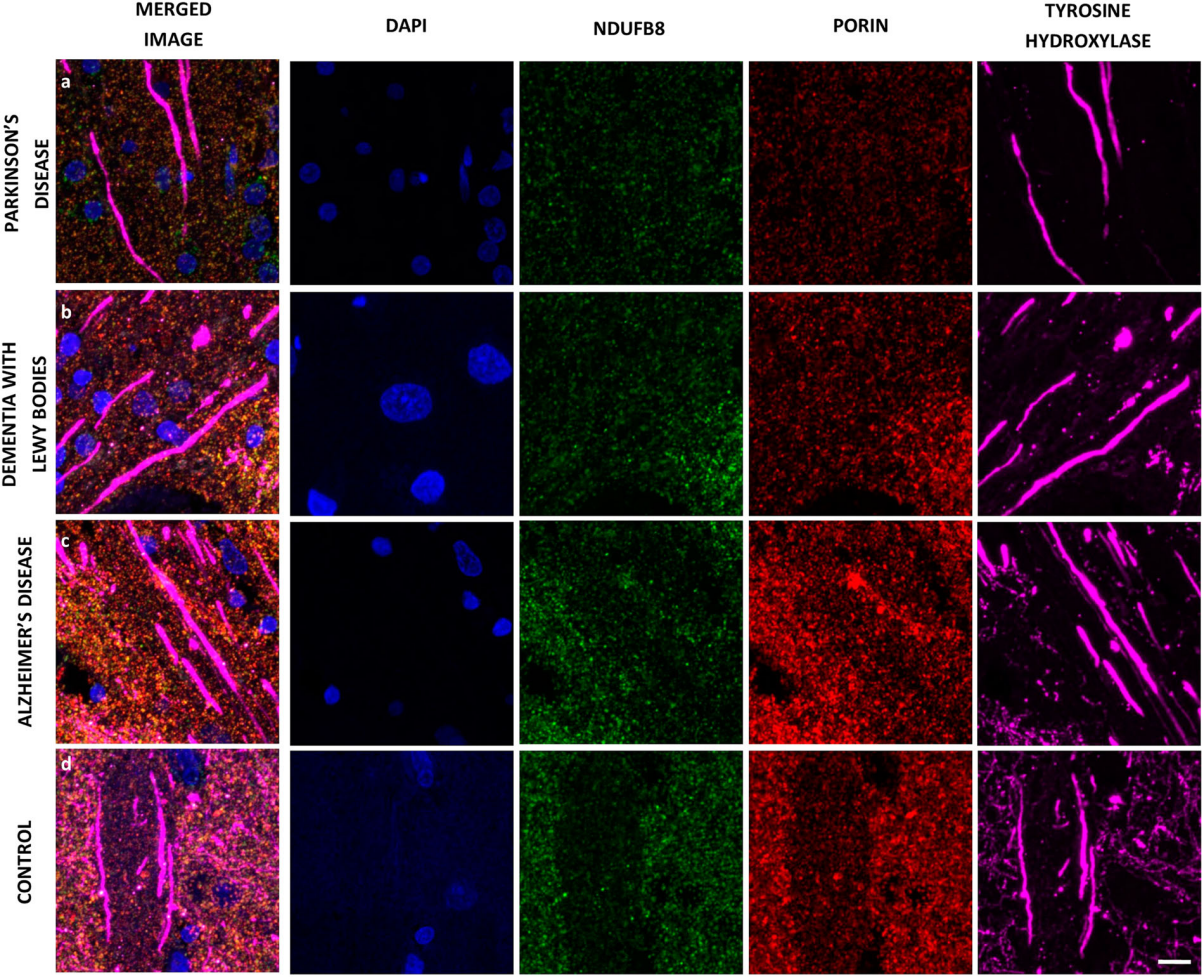
Representative images of immunofluorescence to investigate axonal populations of mitochondria. Triple immunofluorescence for dopaminergic axons (tyrosine hydroxylase), mitochondrial mass (porin) and either mitochondrial complex I or IV was performed on striatal sections from cases with PD (**a**), DLB (**b**), AD (**c**) and Controls (**d**). A nuclear counterstain was used, DAPI. The mitochondria within individual axons could then be analysed using IMARIS analysis software. Scale bar represents 10 µm

Mitochondria are transported to areas of the neuron which exhibit a particularly high energy demand, for example the nodes of Ranvier (to supply ATP for the Na^+^/K^+^ ATPase) and synapses. Therefore we examined whether differences in the density of mitochondria and their expression of mitochondrial complex I and IV existed within surviving dopaminergic axons in Parkinson’s disease (Fig. 3). Such mitochondria may either be stationary or may be in transit, perhaps being retrogradely transported for degradation.

**Fig. 4:**
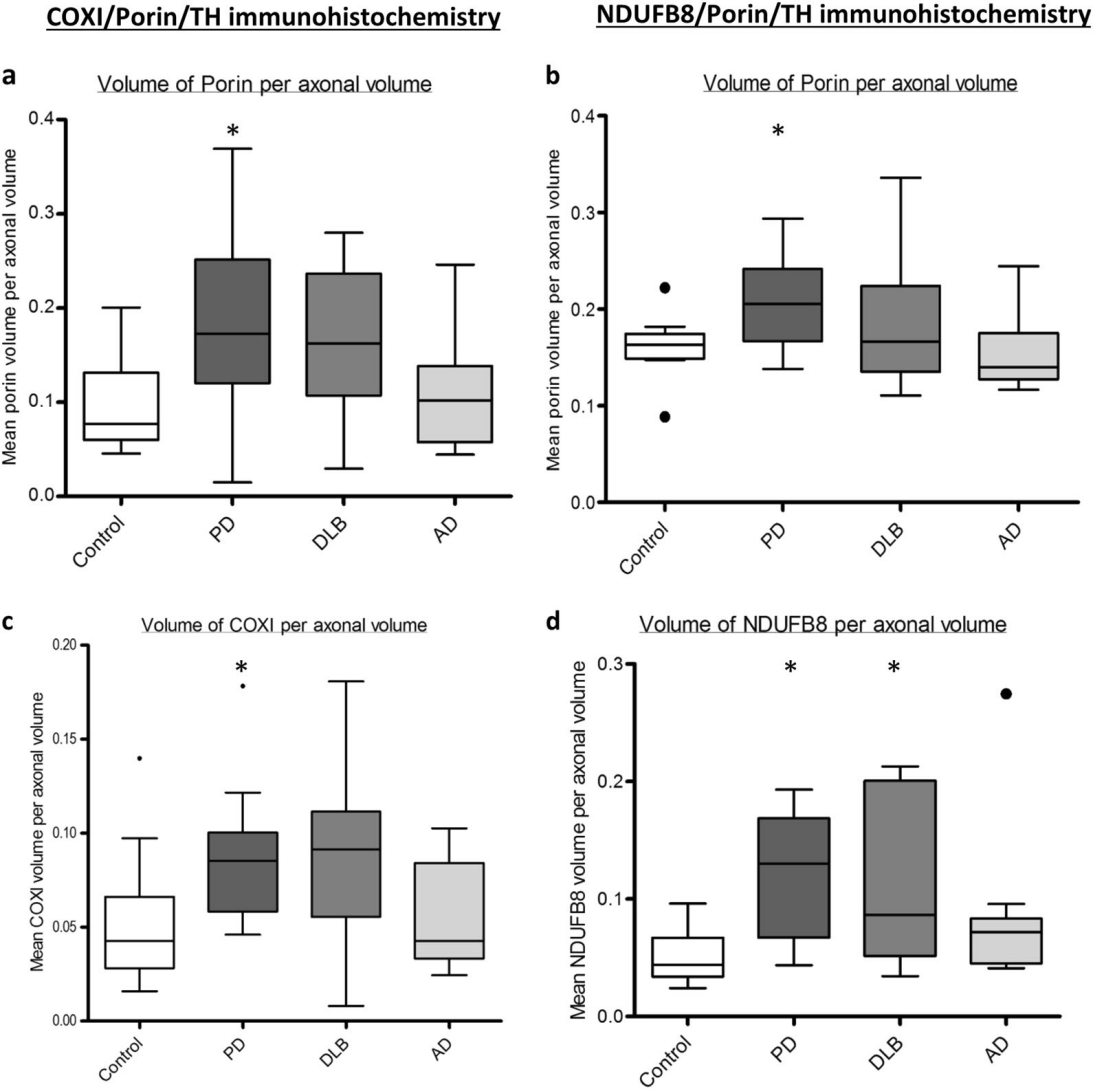
Mitochondrial changes within surviving dopaminergic axons. Mitochondrial density per axon, based upon porin immunoreactivity was highest in cases with PD, when measured in conjunction within both COXI (**a**, *p = 0.0261) and NDUFB8 (**b**, *p = 0.0397). However there was also significant increase in the expression of both COXI (**c**, *p = 0.0359) and NDUFB8 (**d**, *p = 0.0112) in the axons of patients affected by PD. A significant increase in NDUFB8 expression was also detected in dopaminergic axons in DLB (*p = 0.01350)

When the volume of mitochondria which were immunoreactive for porin and either NDUFB8 or COXI were examined we identified significant changes in PD. Firstly, the axons of SN neurons in PD cases showed the highest expression of porin per axonal volume, followed by DLB cases. This suggests that in these situations there is an increase in mitochondrial density per axon. Importantly, significant increases in the expression of NDUFB8 (*p* = 0.0402) and COXI (*p* = 0.04) per mitochondrion were also observed within these axons in PD (Fig. 4a, b). DLB cases also showed a higher expression of these proteins within axonal mitochondria compared to controls, but this was not significant. Expression of these essential proteins in AD axons was similar to the expression in controls as expected (Fig. 4a, b). In addition, there was evidence of mitochondria that were deficient for both NDUFB8 and COXI, and while the percentage of mitochondria that were deficient for these key proteins was reduced in axons in both PD and DLB cases this was not significant (data not shown).

### Mitochondrial alterations within the synapses of dopaminergic neurons

In addition to investigating mitochondrial changes within axons we also examined changes within dopaminergic pre-synaptic terminals in the striatum (Supplementary figure 3). Using a similar approach as detailed in supplementary figure 1 we detected mitochondria located specifically within selected dopaminergic synapses based on two parameters, those which contained mitochondria (based upon porin immunoreactivity) and those for which the DAT volume entirely encapsulated the mitochondria. Once a subset of synapses had been detected the synaptic surface was masked onto either the COXI or NDUFB8 signal and further surfaces were created based on the expression of these proteins. Therefore for each individual synapse we could measure synaptic volume, mitochondrial volume and the expression of mitochondrial complex I or IV.

**Fig. 5:**
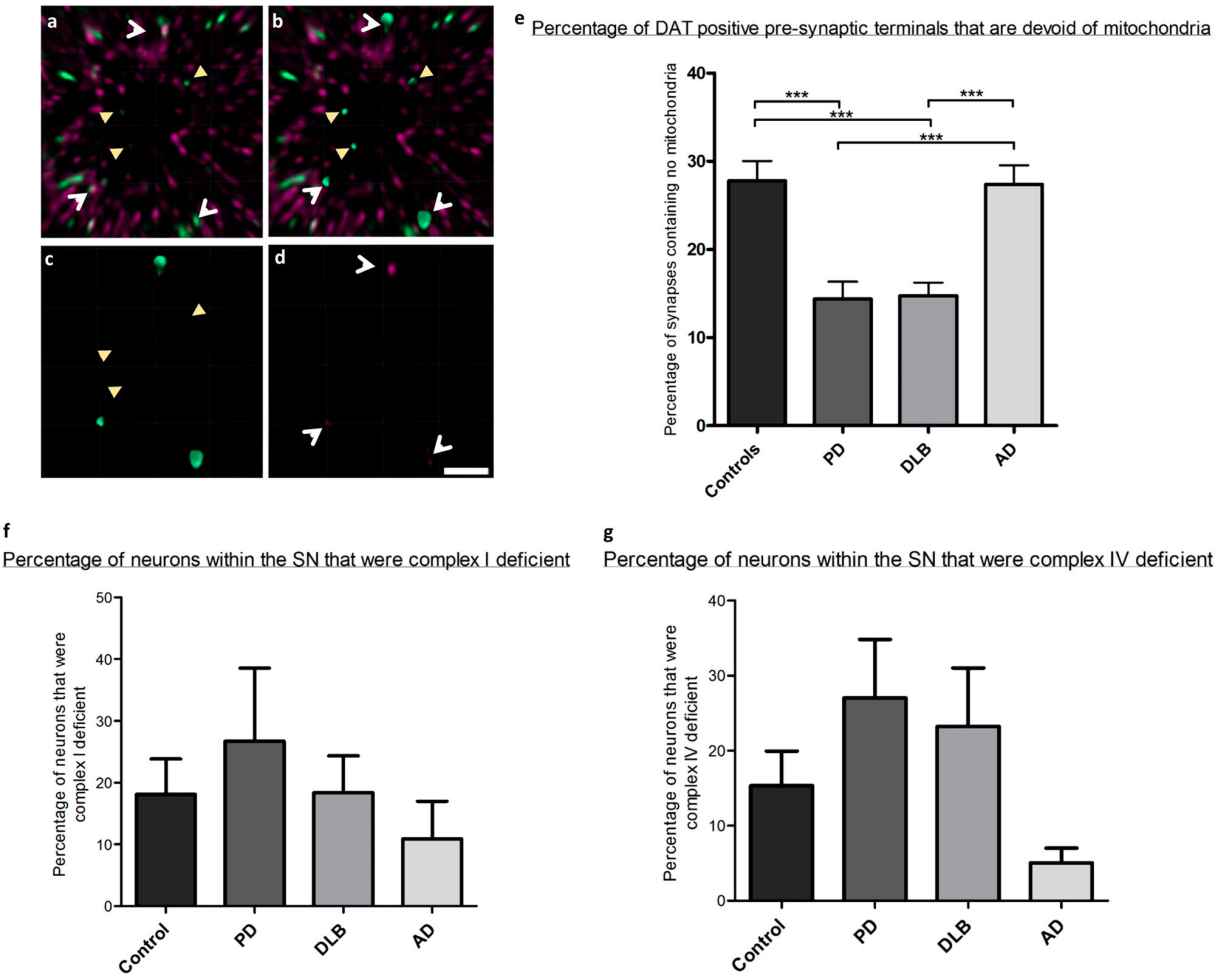
Analysis of empty synapses and respiratory deficiencies within the soma of SN neurons. Using triple immunofluorescence synapses containing no mitochondrial signal were detected. Within a population synapses which contained mitochondria (white, open arrowhead) were found alongside those with no mitochondria (yellow, closed arrowhead) (**a**). Surfaces for both types of synapse can be created (**b**) and when these surfaces are mapped onto the signal for porin it becomes clear that these empty synapses are deficient for porin (**c**) compared to their counterparts (**d**). **e** The percentage of synapses that were devoid of mitochondria was then calculated in each group of patients, there was a significant reduction in the number of these synapses in PD and DLB (***p = ≤0.001). Using quadruple immunofluorescence for TH, COXI, NDUFB8 and porin, the percentage of neuronal cell bodies within the SN that were deficient for NDUFB8 (**f**) or COXI (**g**) could be calculated. The number of cell bodies which were deficient for these proteins was found to be increased in PD and DLB, but not significantly. Scale bar represents 3 µm. Kruskal–Wallis one-way ANOVAs were performed with Dunn’s multiple comparison testing to ascertain statistical significance. Error bars represent s.e.m

When mitochondrial synaptic populations were analysed, no significant differences were detected in either the mitochondrial density or the expression of mitochondrial complexes I and IV within dopaminergic synapses of the striatum. Interestingly we detected empty dopaminergic pre-synaptic terminals that appeared devoid of mitochondria in all groups (Fig. 5a–d). When the percentage of empty synapses was calculated it was found that there was a significant reduction in the proportion of these empty synapses in PD and DLB cases compared to controls and AD cases (*p* ≤ 0.001) (Fig. 5e). A significant relationship (*p* = 0.04) was found between the level of Complex I deficiency within the SN and the number of empty synapses in PD, such that the greater the deficiency for complex I within the cell body the more empty synapses were detected within the striatum (Supplementary figure 4). The number of synapses which were complex I or IV deficient did not differ between the four groups. Furthermore, there was no correlation found between the percentage of synapses which were devoid of mitochondria and overall pre-synaptic terminal number which may have suggested a contribution to the degeneration of these synapses.

### Are alterations in the axonal and synaptic mitochondrial populations correlated with respiratory deficiency in the soma?

Although in human tissue it is not possible to compare cell body changes to synaptic/axonal changes within the same neuron, a measure of respiratory chain deficiency was made within the soma of SN neurons for most of the cases included in this study (with the exception of control 7, AD9, DLB8 and PD3). Intensity measurements were made for immunoreactivity for NDUFB8, COXI and porin within TH positive cell bodies within the SN. A reduction in expression of either of these proteins was defined as an intensity value that had a *z* score of less than −1, normal neurons had a score of between −1 and 1, while an increase was defined as having a score over 1. These parameters were set based on the control data.

There was an increase in the percentage of cell bodies showing reduced expression of both complex I and complex IV in PD cases compared to other groups, and in DLB cases there was an increase in complex IV deficient neurons, however neither of these changes was significant due to the variability between cases (Fig. 5f, g). In addition to measuring deficiency within the neurons of the SN, mitochondrial density was also quantified. Using the signal intensity for porin immunoreactivity we categorised each neuron based on the *z* score for porin. We again found that there was no significant difference between the mitochondrial mass in cell bodies between any of our patient groups (data not shown).

Additionally, we found no correlation between the level of mitochondrial deficiency within the SN and the changes detected in the axonal mitochondrial population or that it drove the mitochondrial population size within the synapse.

To determine if any changes in mitochondrial function in cell soma were associated with changes in lysosomal function or mitophagy, LAMP2A staining was used to determine lysosomal mass in PD. When analysing all neurones within the SN, no overall change in lysosomal mass was seen between PD and control cases (data not shown). When analysing only cells showing mitochondrial deficiency, LAMP2A levels in individual neurones in PD and controls did however correlate with mitochondrial deficiency, with neurones showing reduced levels of complex I expression showing reduced LAMP2A levels (*p* < 0.001; Supplementary Figure 5). PD neurons in general showed higher levels of LAMP2A than control neurons showing equivalent levels of complex I deficiency (*p* = 0.0081; Supplementary figure 5).

### The relationship between synaptic changes and cell body loss within the substantia nigra

To understand whether the synaptic changes detected were related to the degeneration of SN neurons we quantified the number of cell bodies within the SN and investigated the relationship between synaptic alterations and the amount of cell loss. As expected a significant reduction in cell number was seen in PD (*p* = ≤ 0.01) and DLB cases (*p* = < 0.05), compared to both control and AD cases (Supplementary figure 6a). Furthermore, we investigated whether any correlation existed between SN cell body loss and either the prevalence of empty dopaminergic synapses (Supplementary figure 6b) within the striatum or with the pre-synaptic volume (Supplementary figure 6c). No significant relationships were found in either instance. In general, in PD and DLB there was more cell loss seen in cases with fewer empty synapses and this relationship fitted the data most strongly for the PD cases, while a general trend was seen for the pre-synaptic volume to be increased in cases with severe cell loss.

## Discussion

Mitochondrial distribution within neurons is essential to facilitate the provision of ATP to regions of the cell that are particularly energy demanding such as the synapses. At synapses mitochondria provide ATP and are also crucial for Ca^2+^ buffering. Previous studies have shown that mitochondrial populations within axons and synapses can be altered to compensate for pathological changes, for example, during demyelination in multiple sclerosis^35^ or may be disturbed by pathological changes such as amyloid-β.^36,37^ While in mice with a knockout of a key complex I subunit (NDUFS4) neurodegeneration has also been found to be driven by respiratory deficiency within pre-synaptic terminals, including within the brainstem.^38^ Furthermore disruption of mitochondrial fission through the knockout of DRP1 causes dopaminergic neuronal degeneration associated with a severe reduction in mitochondrial mass within the axons of SN neurons.^39^ Therefore given the evidence to suggest that neurodegeneration of dopaminergic synapses may occur prior to the loss of cell bodies within the SN in PD, we wanted to establish if there were any changes in the mitochondrial populations within the axons and synapses of SN neurons that indicated degeneration or compensatory events. A disruption of these populations or an increase in mitochondrial deficiency within them may give further clues to the contribution of mitochondrial dysfunction to the pathogenesis of PD.

### Synaptic and axonal alterations in Parkinson’s disease

We found that there was no difference in either the volume of individual mitochondria or their expression of key electron transport chain proteins, within dopaminergic synapses in PD compared to age matched controls and Alzheimer’s disease cases. While we confirmed a loss of dopaminergic terminals in PD and DLB, the remaining pre-synaptic terminals were enlarged in PD compared to those in controls. This suggests that in PD and DLB pre-synaptic terminals expand with consequent increases of mitochondrial numbers to compensate for the loss of neighbouring synapses and that in the presence of neurodegeneration the mitochondrial populations are maintained within single synapses. The overall effect of this would be a compensatory attempt to maintain synaptic transmission in remaining neurons and to support continued communication with striatal neurons.

We also examined the mitochondrial populations within the axons of SN neurons. Our analysis revealed that total mitochondrial volume was increased in axons and that the expression of mitochondrial complex I and IV subunits was significantly increased per mitochondrion in PD compared to controls and Alzheimer’s disease cases. An increase in electron transport chain proteins would suggest that these mitochondria are producing more ATP to support the maintenance of electrical excitability in these cells even in the presence of neurodegeneration. One possibility however, is that the increase in mitochondrial volume within axons is due to a defect in degradation, potentially due to defective mitophagy.^40,41^ Elevated mitochondrial volumes may be due to decreased mitophagy leading to a build-up of damaged mitochondria within axons and synapses. However, the mitochondria within the axons and synapses do not appear to be grossly altered, showing normal expression of respiratory chain complex subunits similar to control populations and, importantly, similar levels of complex deficiency. This suggests that, at least within axons and synapses, the increased mitochondrial volume is due to increased production rather than a defect in degradation. Within cell bodies we also do not observe increased mitochondrial density suggesting a build-up, rather than an increase in defective mitochondria showing respiratory deficiency. This may indicate that potentially defective mitochondria are not being removed by mitophagy. To explore this we used LAMP2A as a lysosomal mass marker which showed no change in overall lysosome mass between PD and controls. Mitochondrial deficiency did however correlate with reduced LAMP2A expression. This agrees to an extent with evidence that indicates lysosomal depletion occurs in PD SN neurons which may be due to ROS derived from defective mitochondria,^42^ however in rodent models of PD increased mitophagy is a feature.^43^ The reduced energy supply due to mitochondrial deficiency may lead to reduced lysosome production in deficient neurones suggesting lysosomal deficiency in single neurons is due to energy deficiency. The current findings in cell bodies of respiratory deficient SN neurones may indicate reduced mitophagy, although further work will be needed to define this.

The axonal mitochondrial population is a dynamic one. Mitochondria are transported both anterogradely and retrogradely along axons to be either delivered to sites where they are required or to be degraded. A dysregulation or failure of mitochondria to be correctly transported, especially in the presence of respiratory dysfunction, could lead to detrimental effects which may further contribute to neurodegeneration. When we studied the mitochondrial population within the cell bodies of these neurons we found that despite an increase in respiratory deficiency in PD and DLB there was no change in the mitochondrial density, again suggesting that in the face of mitochondrial dysfunction the neuron maintains the mitochondrial population as a compensatory response to maintain neuronal function. A recent study examining age related degeneration of retinal ganglion cells has shown that prior to neurodegeneration mitochondrial transport declines and areas appear within axons that are devoid of mitochondria.^44,45^ Furthermore, the ablation of Milton, an important mitochondrial transport protein, in *Drosophila* photoreceptors leads to alterations of synaptic transmission and an increase in synapses which are devoid of mitochondria, which drives the degeneration of photoreceptors in these flies.^46^ The increased mitochondrial population of surviving axons observed in this study may be due to the presence of increased numbers of damaged synaptic mitochondria being transported back to the cell body for degradation. However we also detected an increase in electron transport chain protein subunits that have previously been shown to correlate with mitochondrial function. Alternatively, given the finding of enlarged synapses with maintained mitochondrial populations in PD, the increase in axonal mitochondria may represent part of a compensatory process where newly derived mitochondria en-route to dopaminergic synapses to maintain synaptic function. Methods to detect mitochondria targeted for degradation will be required to resolve this issue.

We also found that we could detect a population of synapses which appeared devoid of mitochondrial proteins in the normal and pathological brain, the number of which was reduced in DLB and PD compared to controls. An observation that has previously been detected in cultured neurons and hippocampal slices.^47–49^ The purpose and functionality of these empty synapses is unclear as although synaptic neurotransmission has a high mitochondrial and glycolytic requirement for ATP,^34^ it is unlikely that these empty synapses would be functional for long periods of time. It is possible that under normal circumstances these empty synapses might represent a reserve pool which can be called in to use in periods of high functional demand. Given our findings of enlarged synapses, increased axonal mitochondria, and proportional reductions of empty synapses in PD and DLB, this may again be a compensatory attempt to maintain synaptic dopaminergic transmission in the face of neurodegeneration and dopamine deficiency. Previous research has shown that 6-OHDA lesions cause axonal degeneration with an associated loss of synaptic terminals, however following degeneration there is evidence of compensatory axonal sprouting followed by recovery of striatal synapses.^50^ Indeed, our preliminary studies in the 6-OHDA rat model indicate similar changes in synaptic mitochondria to those seen in PD and DLB tissue (Reeve et al., unpublished). If a specific amount of dopamine release from SN synapses is needed to modulate the firing of striatal interneurons, it could be argued that sufficient dopamine must be released under normal circumstances from either a small number of SN neurons, or that only low levels of dopamine are required to effect a change in striatal interneuron firing. Compensation over the suggested 5–10 year prodromal period^2^ might lead to either increases in the number of synapses to effect the same striatal response or to increased synaptic activity in the remaining synapses. Factors which lead to synaptic dysfunction and axonal targeting might therefore alter the response of SN neurons to progressive degeneration in PD. What is of major interest is how this process of reducing the number of empty synapses is regulated, as the identification of the systems which govern this process may be targets for therapeutic intervention to support increased levels of neurotransmission in PD.

Given that this a post mortem study, we can only examine the changes that have occurred by the end stage of the disease process, thus it is possible that the neurons that have been lost are those with mitochondrial dysfunction within their synapses. However, the identification of similar results in DLB cases with intermediate levels of SN loss (and a milder parkinsonian phenotype^51,52^), suggest that the findings are of an early compensatory change. The data presented here supports the hypothesis that remaining SN neurons within the nigrostriatal pathway attempt to compensate for the loss of neighbouring cells by increasing their synaptic volumes and by increasing mitochondrial populations to support continued neural transmission. The reduction in the prevalence of empty synapses in PD and DLB could suggest their recruitment in PD, as the data from Stowers et al. (2002) might suggest,^46^ which would support the increase in Milton controlled trafficking of mitochondria. The toxin MPTP causes mitochondrial dysfunction through the inhibition of mitochondrial complex I, but in model systems it has also been shown to inhibit the transport of mitochondria within axons leading to synaptic loss.^53^ An ability to transport functional mitochondria through the Milton/Miro pathway appears crucial for neuronal and synaptic survival. Finally, many neurons within the SN accumulate mitochondrial dysfunction with advancing age.^22^ Therefore one hypothesis would be that to maintain neuronal function, and since ATP is required for axonal transport, functional mitochondria should be mobilised along axons and to synapses. In either scenario the axons and synapses studied are those which have survived longest and the argument could be made for these changes in synapses and mitochondria to be protective. If this is a general compensatory neuronal response mechanism to increase synaptic function following degeneration is however, unclear. Different neuronal populations are affected in diverse neurodegenerative disorders and it would be of interest to determine if specific neurones in other brain regions affected by neurodegeneration respond in the same way.

## Conclusions

Despite significant neuronal and synaptic loss in PD, dopaminergic neurons maintain healthy mitochondrial populations within their synapses and axons, with mitochondria showing a normal respiratory chain complement. This along with an increase in the volume of pre-synaptic terminals may represent a compensatory mechanism. Furthermore there is a reduction in the percentage of synapses that are devoid of mitochondria in PD and DLB, suggesting that this is a compensatory mechanism to prevent synaptic dysfunction and loss.

## Methods

### Tissue

All human tissue for this study was obtained from the Newcastle Brain Tissue Resource. Consent for the use of all tissue had been given by the donors or next of kin with permission of the National Health Service Local Research Ethics Committee and use conformed to the UK MRC Guidelines on the use of tissue in medical research. Serial coronal transverse formalin-fixed, paraffin-embedded sections of putamen and transverse upper midbrain were cut at a thickness of 5 µm from 10 PD cases, 10 DLB cases and 11 Alzheimer’s disease cases (included as a disease control) (Table 1). All cases were pathologically and clinically confirmed. These cases were compared to 11 age matched controls with only age-associated pathological changes. Details of the cases used for this study can be found in supplementary table 1.

**Table 1:** Mean age and PM delay for cases used

Disease	Mean age (years)	Age range (years)	Mean PM delay (hours)	Sex
PD	80.8	70–92	46.3 (15–88)	3 female/7 male
DLB	77.5	71–88	39.6 (8–68)	3 female/7 male
AD	83.4	75–88	54.9 (29–82)	5 female/6 male
Dementia with Lewy bodies	80.8	69–89	31.8 (8–64)	6 female/ 5 male

### Immunohistochemistry

Double immunofluorescence was used to measure the volume of dopaminergic synapses within the striatum. To do this we utilised antibodies for the dopamine transporter (DAT) to label pre-synaptic terminals and the dopamine D2 receptor (D2R) to label post synaptic regions. We then utilised triple immunofluorescence to allow the quantification of mitochondrial mass (porin) and mitochondrial respiratory chain proteins (complex I 20 kDa (NDUFB8, C120) or cytochrome *c* oxidase subunit I (COXI)) within either single axons (with tyrosine hydroxylase; TH) or single pre-synaptic terminals (DAT). A final combination of TH, COXI, NDUFB8 and porin, or NDUFB8 and LAMP2A to define lysosomal mass was applied to upper midbrain sections to allow a measure of mitochondrial deficiency to be calculated in the cell bodies of dopaminergic neurons within the SN.

The protocol used was modified from that previously reported.^54^ Briefly, sections were deparaffinised and rehydrated through an ethanol series. Antigen retrieval was performed in 1 mM EDTA (pH 8.0, Affymetrix Inc) at high temperature and with pressure. Non-specific binding was blocked with a 30 minute incubation in 1% normal goat serum (Sigma) diluted in phosphate buffered saline (PBS), which was also used to dilute primary and secondary antibodies. Primary antibodies were applied and incubated for 90 min at room temperature. Following appropriate washes in PBS, fluorescently conjugated secondary antibodies were applied and incubated for 60 min at room temperature (see supplementary table 2 for details). Following PBS washes 2 mM Hoechst (ThermoFisher) was applied and incubated for 30 min to give a nuclear counterstain. Again the sections were washed in PBS before a final 10 min incubation in Sudan black (in 70% EtOH) to quench autofluorescence. After application of Sudan black (BDH) sections were washed rapidly in PBS with 0.1% Tween to remove excess solution and then in PBS. Sections were then mounted using Prolong Gold Antifade (ThermoFisher) and stored at −20 ˚C before imaging.

### Cresyl fast violet (CVF) stain and cell counts

CFV staining was performed to allow dopaminergic neuronal counts to be performed. Three 20 µm thick sections of upper midbrain were cut, stained using CFV (Sigma) and used to count the cell bodies of the dopaminergic neurons.^55^ Two-dimensional cell counts were performed as described previously.^55,56^ The SN was outlined at a low magnification and a meander scan through this region performed. Neurons were counted that contained neuromelanin and a clearly defined nucleus. The cell count for each section was then calculated as a percentage of the mean control cell count for all control sections/cases.

### Image analysis and quantification

Sections were imaged using a Nikon A1R Confocal microscope and NiS elements software. *Z*-stack images were taken using a x63 oil immersion objective and the *Z*-stack controlled using a Piezo drive. The settings for laser power, gain and offset were set on ‘no primary’ and single stained controls (whereby one primary was applied followed by the cocktail of secondary antibodies), for each staining run. These settings were then used to image all cases. Image capture and analysis was done blind to disease status. Based on power calculations, six *Z* stack images were captured per case ensuring the capture of a number of synapses or at least two axons per image. Images were then analysed using IMARIS software (Bitplane, Oxford Instruments).

### Synapse morphological analysis

To analyse synaptic volume based on DAT and D2R immunoreactivity, *Z* stacks were deconvolved, imported into IMARIS and resized according to their original dimensions (Supplementary figure 1a). Surfaces were created for DAT positive pre-synaptic terminals (green) and D2R positive post-synaptic terminals (Red) (Supplementary figure 1b). Once these surfaces had been created (Supplementary figure 1c), synapses were identified as locations where the green and red surfaces touched (Supplementary figure 1d). These synapses were then analysed for total synaptic volume as well as pre and post-synaptic terminal volume. The number of pre and post-synaptic terminals per unit area was also recorded.

### Axonal analysis

Each *Z* stack was deconvolved, imported into IMARIS and resized depending on its original dimensions (Supplementary figure 2a). The axonal tyrosine hydroxylase staining was selected (the 647 nm channel, purple) and a surface was created based on this stain (Supplementary figure 2b & c). The length and volume of each axon was recorded in order to allow expression of measured metrics per unit axonal length or volume. This axonal mask was applied onto the porin channel (546 nm, red), which allowed the mitochondria located within axons to be selected (Supplementary figure 2d). Once these mitochondria had been selected a second surface was created for the porin positive mitochondria, based on no primary controls the staining intensity boundaries were set between 500 and 4095 (the saturation intensity), while the smoothness was set at 0.1 and the background subtraction at 0.5. The process was then repeated for either NDUFB8 or COXI (488 nm channel, green), with the intensity boundaries between 400 and 4095 (Supplementary figure 2e). Each axon was selected in turn and the volume and number of mitochondria positive for porin and NDUFB8 or COXI was analysed (Supplementary figure 2f).

### Synaptic mitochondrial analysis

Each *Z* stack was deconvolved, imported into IMARIS and resized depending on its original dimensions. The synaptic terminal staining (DAT, 488 nm, green) was selected and a surface was created. The smoothness was set at 0.1 and the background subtraction at 0.75, while the limit of fluorescence intensity was set at 4095. The volume of each synaptic terminal was recorded and used to express measured metrics per unit synaptic volume. This mask was applied onto the porin channel (647 nm, purple), which allowed the mitochondria located within synapses to be selected. Based on no primary controls the staining intensity boundaries were set between 500 and 4095, while the smoothness was set at 0.1 and the background subtraction at 0.5. To avoid biased selection a subset of synapses were chosen for which the porin immunoreactivity was encapsulated completely within the DAT surface. Once these synapses had been selected, they were masked onto the NDUFB8 or COXI channel (546 nm channel, red) and a third surface was created for the respiratory chain proteins, with the intensity boundaries between 400 and 4095. Each synapse was then analysed in turn and the volume of Porin and NDUFB8 or COXI positive mitochondria was recorded.

### Cell body analysis

To allow a measurement of mitochondrial deficiency within the cell bodies of SN neurons we performed quadruple immunofluorescence as described above. Twenty five, single plane images were taken per case at x40 magnification. Neurons were identified by the presence of neuromelanin, a defined nucleus and a positive signal for TH. The area of each cell body was then defined and the signal intensity per unit area was measured. Measurements from no primary controls were also taken and these values subtracted from the intensities gathered from positively stained neurons. Similarly, we measured complex I deficiency in neurones using NDUFB8 and lysosomal mass using LAMP2A and the intensity per unit area determined.

### Statistical analysis

For all data analyses, Box Cox regression was used when appropriate to identify optimal data transformation to achieve normality. Additional linear regression, *t* tests or one-way ANOVAs were used where appropriate for statistical testing. For the measurement of deficiency within the cell body, the intensities were log transformed and normalised to porin, *Z* scores were then calculated.

### Data availability statement

All data generated or analysed during this study are included in this published article (and its supplementary information files).

## Electronic supplementary material

Supplementary Information(PDF 3056 kb)
