# Supplementary material for: Mitochondrial dysfunction within the synapses of substantia nigra neurons in Parkinson’s disease
Source: NPJ Parkinsons Dis. 2018 Mar 26;4:9. doi: 10.1038/s41531-018-0044-6 (PMC5979968; doi:10.1038/s41531-018-0044-6)
Supplement: Supplementary file 1 — Supplementary Information(PDF 3056 kb) [file 41531_2018_44_MOESM1_ESM.pdf]

Case	Age	Sex	Age of onset	Disease duration (Years)	Diagnosis	Cortical Lewy body score	Neurofibrillary tangle score (Braak stage)
**PD1**	76	F	72	4	PD	10	1
**PD2**	87	F	76	11	PD (with dementia)	-	3
**PD3**	71	M	68	3	PD (MSA)	9	1
**PD4**	80	M	75	5	PD	8	1
**PD5**	76	M	69	7	PD	11	1
**PD6**	81	M	70	11	PD	-	3
**PD7**	85	M	85	10	PD	9	4
**PD8**	90	M	79	11	PD	-	2
**PD9**	92	F	80	12	PD	-	1
**PD10**	70	M	55	15	PD	4	2
**DLB1**	77	M	73	4	Probable DLB	19	2
**DLB2**	75	F	67	8	Clinical DLB	20	1
**DLB3**	75	M	68	7	DLB	6	2
**DLB4**	88	F	80	8	DLB	11	3
**DLB5**	77	M	72	5	Probable DLB	17	3
**DLB6**	71	M	68	3	Probable DLB	18	3
**DLB7**	75	F	72	3	Probable DLB	20	6
**DLB8**	81	M	76	5	Probable DLB	16	3
**DLB9**	79	M	74	5	Probable DLB	10	2
**DLB10**	77	M	74.5	2.5	Clinical DLB	18	2
**AD1**	81	M	77	4	AD	No LB pathology	3
**AD2**	87	F	79	8	AD	No LB pathology	6
**AD3**	81	F	80	1	Possible vascular dementia	No LB pathology	5
**AD4**	84	F	-	-	Probable AD	No LB pathology	6
**AD5**	88	M	86	2	AD	No LB pathology	6
**AD6**	85	M	73	12	AD	No LB pathology	6
**AD7**	82	M	76	6	Probable AD	No LB pathology	5
**AD8**	76	M	73	3	Probable AD	No LB pathology	6
**AD9**	83	F	81	2	AD	No LB pathology	5
**AD10**	87	M	-	-	Probable AD	No LB pathology	6
**AD11**	75	F	72	3	AD	No LB pathology	6
**Control 1**	89	F	-	-	Control	No LB pathology	3
**Control 2**	78	F	-	-	Control	No LB pathology*	0
**Control 3**	88	F	-	-	Control	No LB pathology	3
**Control 4**	83	M	-	-	Control	No LB pathology	2
**Control 5**	87	M	-	-	Control	No LB pathology	2
**Control 6**	89	F	-	-	Control	No LB pathology	2
**Control 7**	77	M	-	-	Control	No LB pathology	2
**Control 8**	69	F	-	-	Control	No LB pathology	1
**Control 9**	78	F	-	-	Control	No LB pathology	2
**Control 10**	75	M	-	-	Control	No LB pathology	1
**Control 11**	73	M	-	-	Control	No LB pathology	0

*Brainstem LB in dorsal motor nucleus of the vagus

Supplementary table 1 - Specific case information - including diagnosis, disease progression, disease duration and neuropathological information. Cortical Lewy body scores are alpha-synuclein positive scores. LB – Lewy body, MSA – multisystem atrophy.

Antigen	*IG subtype*	*Source*	*Dilution*
Tyrosine Hydroxylase (TH)	*Rabbit polyclonal*	*Sigma*	*1 in 100*
Dopamine Transporter (DAT)	*Rat monoclonal*	*Millipore*	*1 in 100*
Dopamine D2 Receptor (D2R)	*Mouse monoclonal(IgG2a)*	*Mitosciences (AbCam)*	*1 in 100*
Mitochondrial complex IV subunit I (COXI)	*Mouse monoclonal(IgG2a)*	*Mitosciences (AbCam)*	*1 in 100*
Porin/VDAC1	*Mouse monoclonal (IgG2b)*	*Mitosciences (AbCam)*	*1 in 100*
Mitochondrial complex I subunit (NDUFB8)	*Mouse monoclonal (IgG1)*	*Mitosciences (AbCam)*	*1 in 100*
LAMP2A	*Rabbit polyclonal*	*AbCam*	*1 in 100*
Goat anti Rabbit IgG, 647 conjugate		*Alexafluor (Life Technologies)*	*1 in 200*
Goat anti Rat IgG, 488 conjugate		*Alexafluor (Life Technologies)*	*1 in 200*
Goat anti Mouse Ig2a, 546 conjugate		*Alexafluor (Life Technologies)*	*1 in 200*
Goat anti Mouse IgG2b, 647 conjugate		*Alexafluor (Life Technologies)*	*1 in 200*
Goat anti Mouse IgG1, 546 conjugate		*Alexafluor (Life Technologies)*	*1 in 200*
Goat anti Mouse IgG1, 488		*Alexafluor (Life Technologies)*	*1 in 200*

Supplementary table 2 – Details of antibodies used for this study.

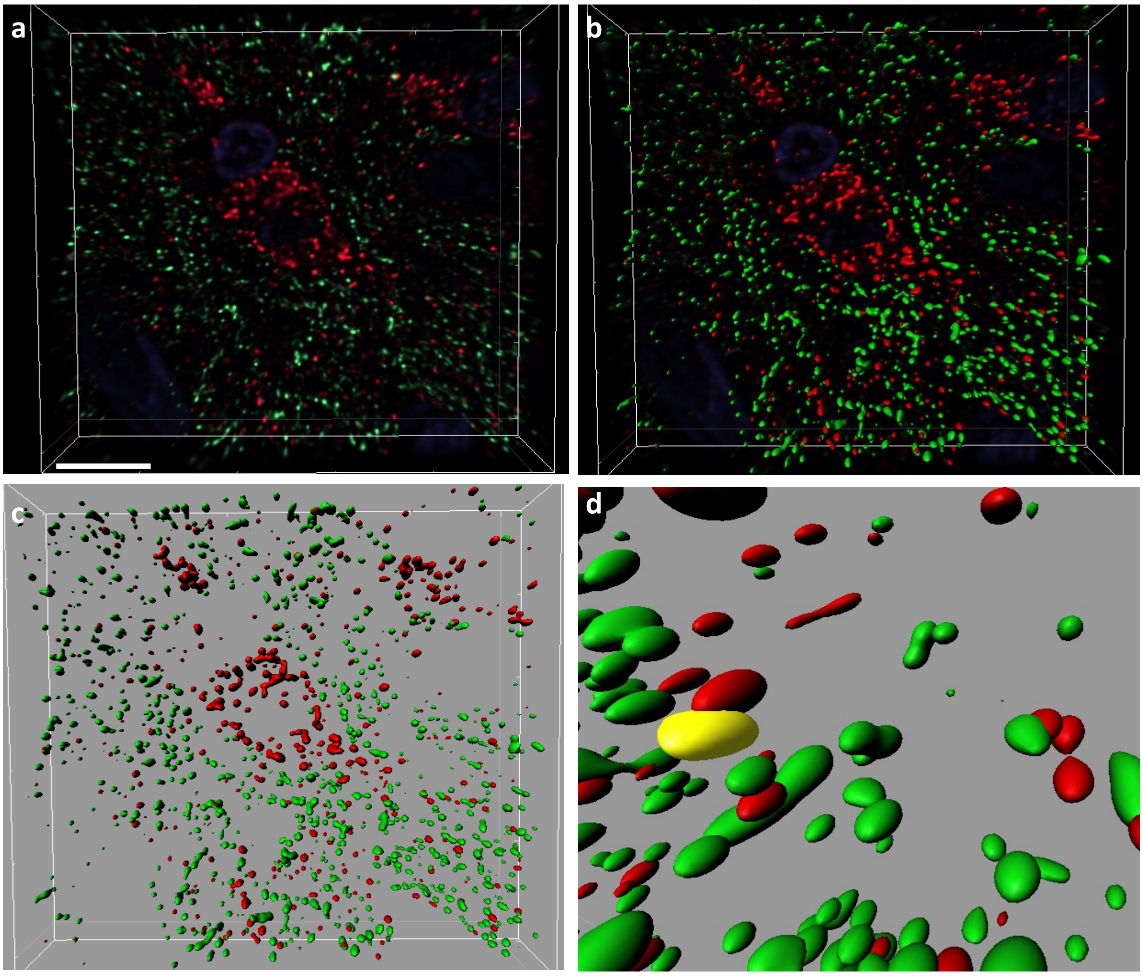

Supplementary figure 1 – Analysis of synaptic volume

To analyse alterations within synaptic volume, the relevant confocal, Z-stack image was imported into IMARIS (A), surfaces were then created based on the fluorescent signal from the DAT immunoreactivity (green, 488nm) and D2 receptor reactivity (red, 546nm) (B). These surfaces then allow measurements of the volume of each structure to be made (C). The volume of synapses were measured where the pre and post synaptic structures were touching (D), an example of such a synapse is highlighted in yellow. Scale bar represents 10μm.

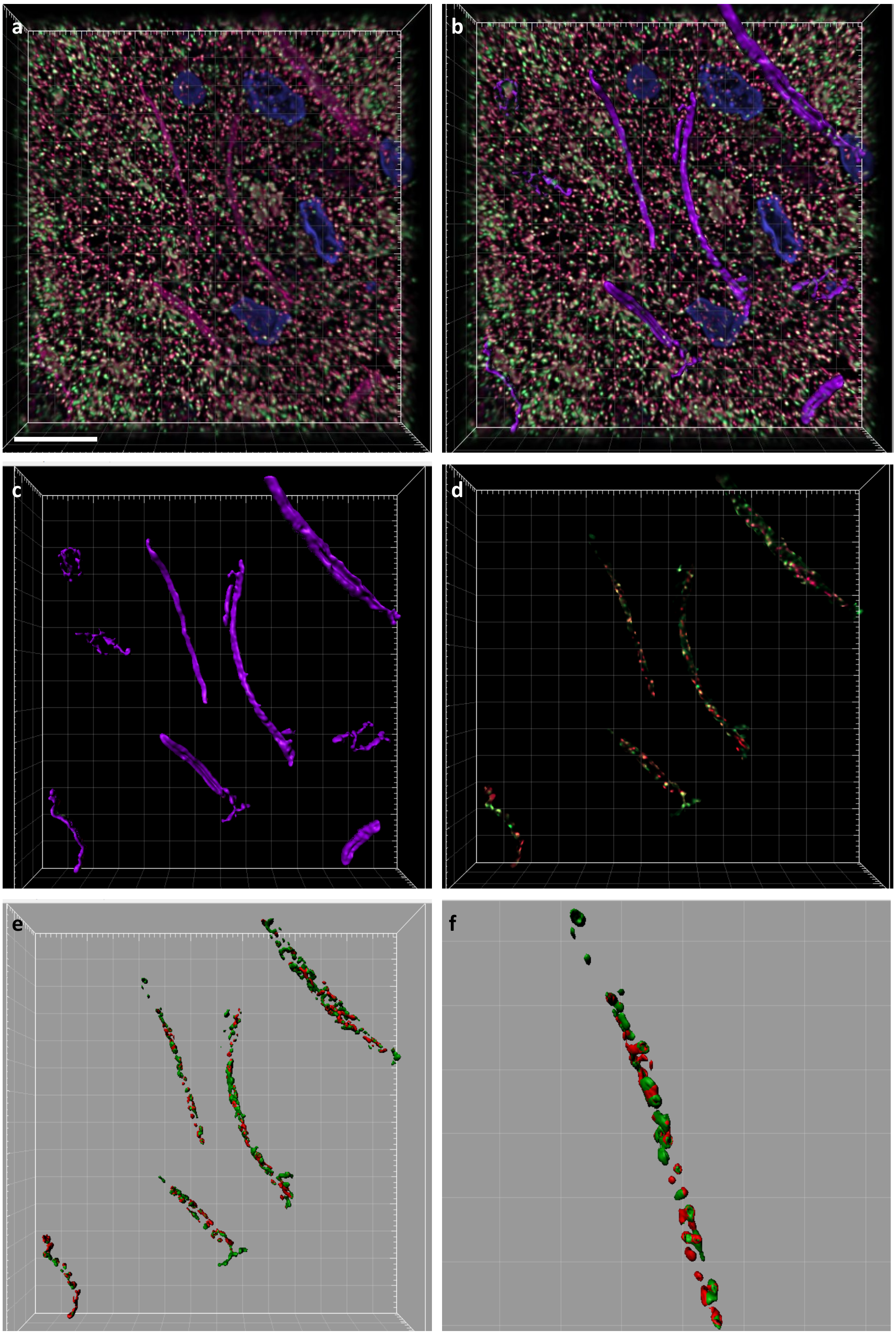

Supplementary figure 2 – Analysis of mitochondrial populations within dopaminergic axons.

Using the IMARIS image analysis package the investigation of individual mitochondria within axons could be performed. Z stack images of triple immunofluorescence were obtained (A). Tyrosine hydroxylase detected using a 647nm conjugated secondary (magenta), Porin detected using 546nm (red), complex I and IV detected using 488nm (green) and nuclei 405nm (blue). Axons were defined based upon tyrosine hydroxylase immunoreactivity and a surface created in 3D (B/C). These axonal surfaces were then used as masks for the mitochondrial channels (D). Mitochondrial surfaces were then created (D) and the volume and number of mitochondria in individual processes could be studied (E & F). Scale bar represents 10µm.

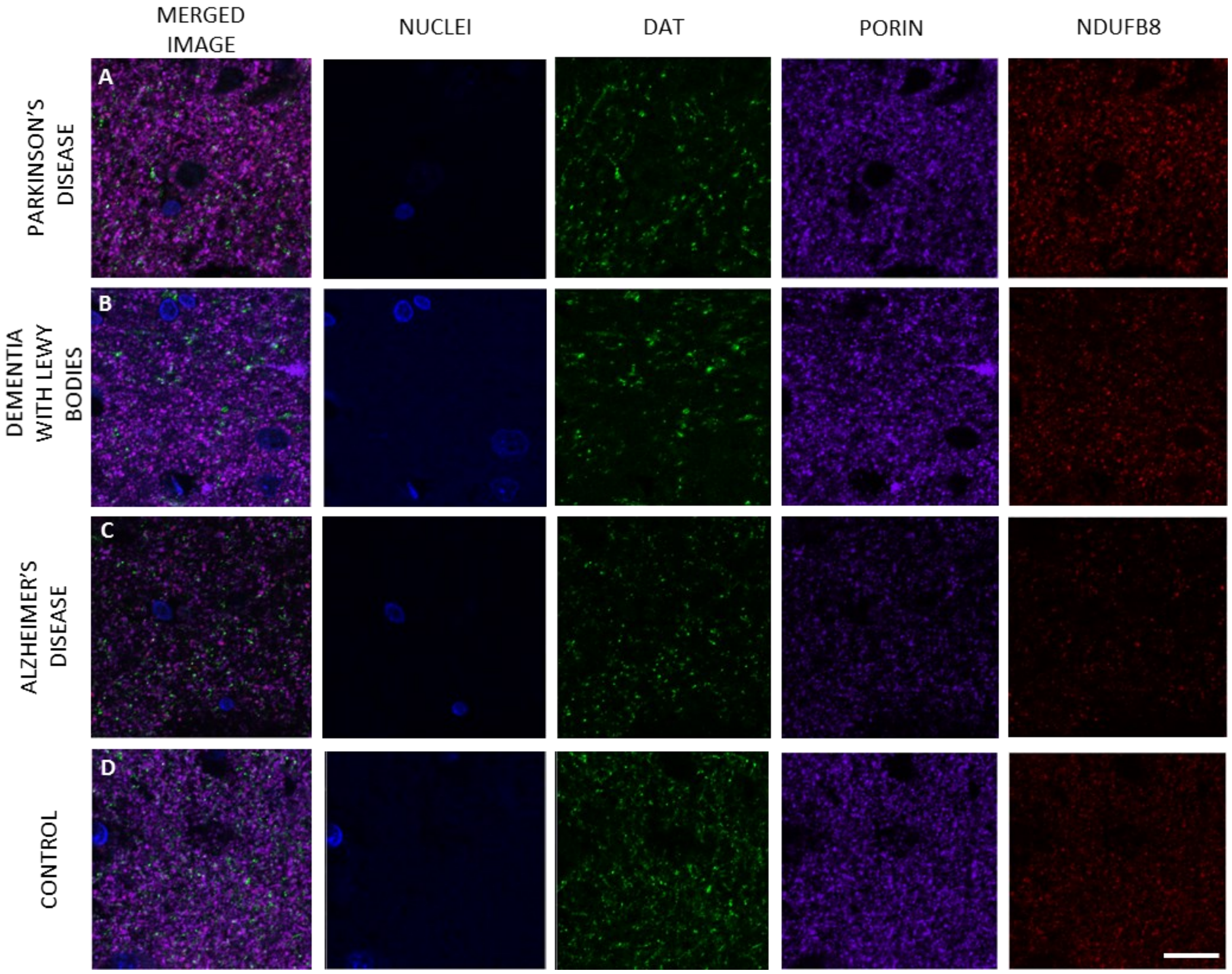

Supplementary figure 3 – Representative images of immunofluorescence to investigate synaptic populations of mitochondria.

Triple immunofluorescence for dopaminergic synapses (DAT – dopamine transporter (488nm)), mitochondrial mass (porin (647nm)) and either mitochondrial complex I (NDUFB8 (546nm) or IV was performed on striatal sections from cases with PD (A), DLB (B), AD (C) and Controls (D). A nuclear counterstain was used, DAPI. The mitochondria within individual synapses could then be analysed using IMARIS analysis software. Scale bar represents 10µm.

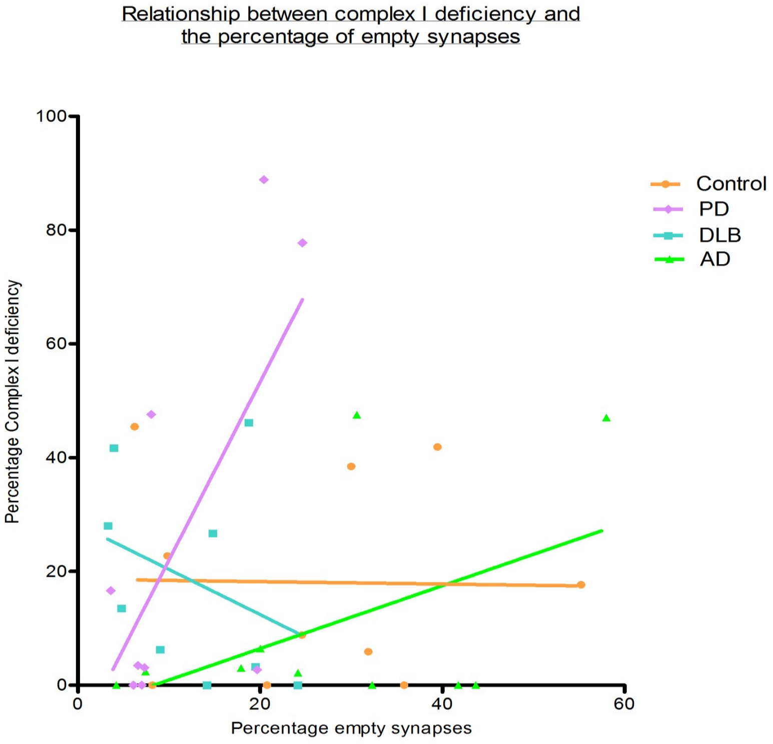

Supplementary figure 4 – Investigating the relationship between mitochondrial deficiency within the cell body and the prevalence of empty synapses.

We investigated if a deficiency for complex I within substantia nigra neuronal cell bodies correlated with the percentage of empty synapses detected in the striatum. No significant correlations were found for complex IV deficiency, but a significant relationship (p=0.04) was found between the level of Complex I deficiency within the SN and the number of empty synapses in PD. Statistical testing using the Spearman correlation coefficient was performed.

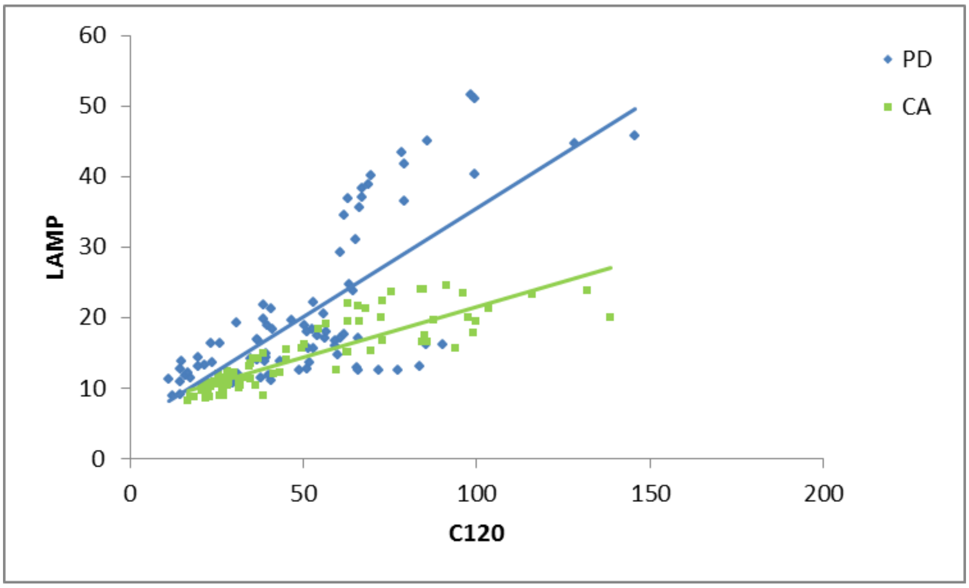

Supplementary figure 5 – Relationship between mitochondrial deficiency and lysosomal LAMP2A levels within substantia nigra neuron cell bodies in PD.

Levels of the lysosomal marker LAMP2A were determined in cell bodies (n=150) of substantia nigra neurons simultaneously stained for LAMP2A and NDUFSB8/C120. LAMP2A positively correlated with NDUFSB8 levels in PD (blue) and control tissue (CA, green) (Spearman $p<0.001$). The relationship of LAMP2A levels and complex I deficiency was significantly different between PD and control cases (gradient $p=0.0081$), with greater LAMP2A at equivalent C120 levels in PD vs. Controls.

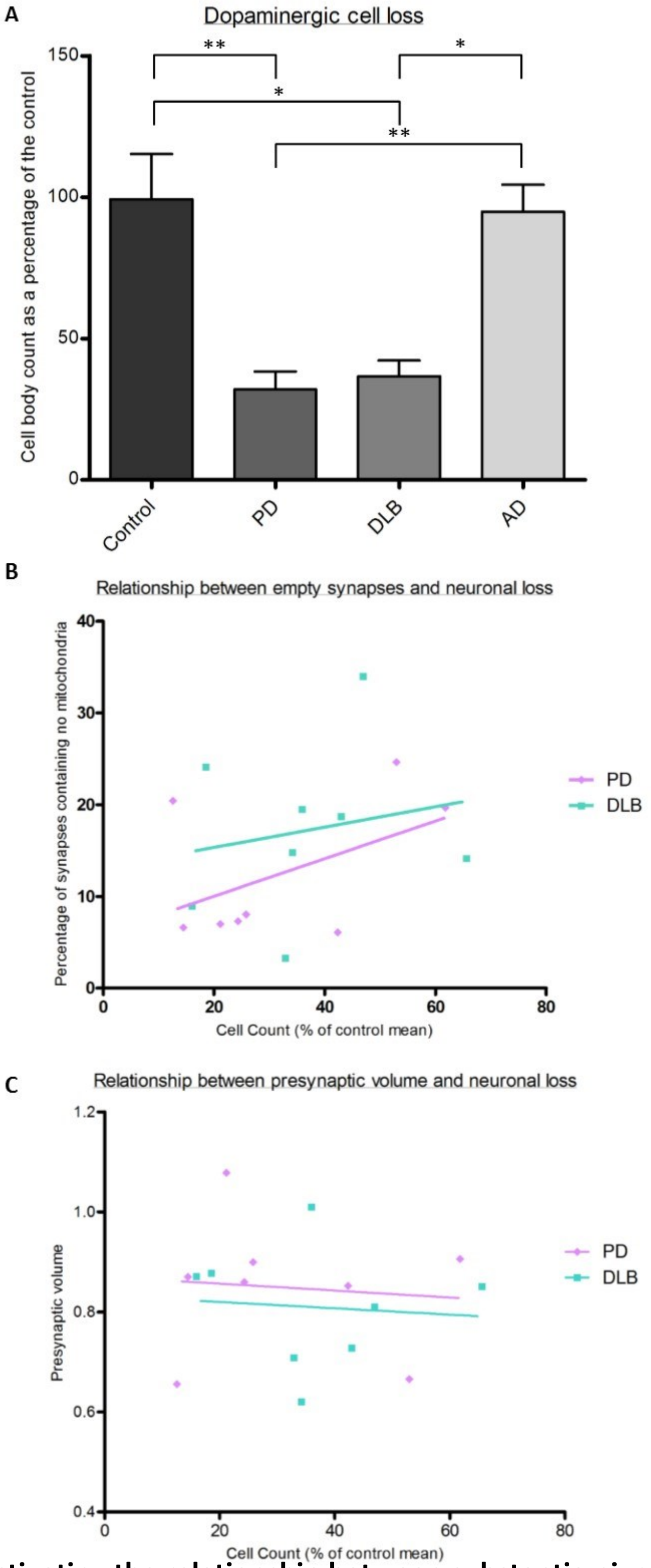

Supplementary figure 6 - Investigating the relationship between substantia nigra neuronal loss and synaptic alterations.

A significant loss of dopaminergic neurons was detected in both PD and DLB cases within the substantia nigra (A). Kruskal-Wallis one-way ANOVAs were performed with Dunn's multiple comparison testing to ascertain statistical significance (*=P<0.05, **=P≤0.01). A further investigation was performed to ascertain if a relationship existed between the proportion of empty synapses within a case (B) or the presynaptic volume (C) and the amount of cell loss. No significant relationships were found. Error bars represent s.e.m.
